# Improvement in mental health following total hip arthroplasty: the role of pain and function

**DOI:** 10.1186/s12891-019-2669-y

**Published:** 2019-06-29

**Authors:** Uyen-Sa D. T. Nguyen, Thomas Perneger, Patricia D. Franklin, Christophe Barea, Pierre Hoffmeyer, Anne Lübbeke

**Affiliations:** 1 Department of Orthopedics & Physical Rehabilitation, University of Massachusetts Medical School, 55 Lake Ave North, Worcester, MA 01655 UK; 2 Department of Biostatistics and Epidemiology, University of North Texas Health Science Center, School of Public Health, 3500 Camp Bowie Blvd, Fort Worth, TX 76107 USA; 3 Clinical Epidemiology Service, Geneva University Hospital, Rue Gabrielle-Perret-Gentil 4, CH-1211 Geneva, Switzerland; 4 Department of Medical Social Science, Northwestern University Feinberg School of Medicine, 633 St. Clair St, 19th floor, Chicago, IL 60611 USA; 5 Department of Orthopaedic Surgery, Geneva University Hospital, Rue Gabrielle-Perret-Gentil 4, CH-1211 Geneva, Switzerland

**Keywords:** Total hip arthroplasty, Mental health, Pain, Physical function, SF-12, WOMAC

## Abstract

**Background:**

Mental health has been shown to improve after total hip arthroplasty (THA). Little is known about the role of pain and function in this context. We assessed whether change in mental health was associated with improvement in pain and function 1 year post-surgery.

**Methods:**

This prospective study included patients enrolled in a THA registry from 2010 to 2014. We examined the mental component score (MCS) before and 1 year post-surgery, and 1-year change, in association with Western Ontario McMaster Universities (WOMAC) pain and function scores. All scores were normalized, ranging from 0 to 100 (larger score indicating better outcome). Analyses were adjusted for potential confounders.

**Results:**

Our study included 610 participants, of which 53% were women. Descriptive statistics are as follows: the average (SD) for age (years) was 68.5 (11.8), and for BMI was 26.9 (4.9). In addition, the MCS average (SD) at baseline was 44.7 (11.2), and at 1-year after THA was 47.5 (10.5). The average change from baseline to 1-year post-THA in MCS was 2.8 (95% CI: 1.9, 3.6), for an effect size of 0.26. As for the WOMAC pain score, the average change from baseline to 1-year post-THA was 44.2 (95%CI: 42.4, 46.0), for an effect size of 2.5. The equivalent change in WOMAC function was 38.1 (95% CI: 36.2, 40.0), for an effect size of 2.0. Results from multivariable analysis controlling for covariates showed that an improvement of 10 points in the 1-year change in pain score resulted in a 0.78 point (95%: CI 0.40, 1.26) increase in the 1-year change in MCS, whereas a 10-point improvement in the 1-year change in function was associated with a 0.94 point (95% CI: 0.56, 1.32) increase.

**Conclusions:**

Mental health significantly improved from baseline to 1-year post-THA. Greater improvement in pain and function was associated with greater improvement in mental health 1 year post-THA.

## Background

Poor mental health is reported among patients with pain and functional disability [1–3], and is common in osteoarthritis (OA) patients. In fact, osteoarthritis and mental health disorders are the leading causes of disability in older adults [4–6]. Depressive symptoms in people with hip OA were higher (23–34%) compared with other chronic diseases such as diabetes, coronary heart disease or cancer (16–24%) [7]. More specifically, depressive symptoms were found in 34% of OA patients on a waiting list for total hip arthroplasty (THA), and in 23% of patients waiting for total knee arthroplasty (TKA) [7]. Pain is thought to affect subsequent mood through its effect on disability [8, 9]. Because of the inter-relation among pain, disability and mental health status, the improvement in pain and function resulting from total joint arthroplasty (TJA) may also result in improvement in mental health status [10].

Previous research on mental health status in OA concentrated either on assessing mental health status before and after TJA [11–16] [17–22] or on evaluating how the presence of poor preoperative mental health or of depression impacted postoperative pain and function [7, 23–25], [26–28]. However, to the best of our knowledge, the relationship between degree of pain and function improvement and mental health improvement after THA has not been well studied. Therefore, the aims of our current study were: 1) to examine the change in mental health from before to 1 year after surgery and to identify variables associated with improvement; and 2) to examine the association between change in mental health and change in pain and function over time.

## Methods

### Study population

As part of a prospective THA cohort that began in 1996 at a large public hospital in Switzerland, data before and after surgery were systematically collected on all THAs performed at the institution [29]. For this current longitudinal study, all elective primary THAs (and no further contra-lateral hip arthroplasty during the follow-up year) operated at the Orthopedic Department between January 1 and December 31, 2010, and between January 1, 2012 and July 31, 2014 were eligible. Data from 2011 were not included because preoperative questionnaires had not been sent out routinely during that year. All eligible THA patients (*n* = 1045) received questionnaires, which were sent between 10 and 14 days prior to surgery. Of those, 848 questionnaires were returned (81.1%). One year after surgery follow-up questionnaires were sent to all eligible patients, to which 785 (75.1%) responded. Overall, 636 (60.9%) of the eligible patients with THA responded to both the preoperative and the 1-year postoperative questionnaire, with 610 people having data on mental health status and pain or function data and were included in this study.

### Study instruments

At baseline and 1 year after surgery, patients completed patient-reported outcomes using questionnaires. Mental health status was assessed using the mental component score (MCS) of the Medical Outcomes Study Short Form-12 (SF-12) [17], which is a generic health-related quality of life measure. Pain and function were assessed using the reduced form of the Western Ontario McMaster Universities (WOMAC) [30], which is a disease-specific instrument for the assessment of osteoarthritis of the hip and knee. The WOMAC pain and function scales were normalized to a range of 0 (lowest possible score) to 100 (highest possible score), with an increasing score indicating better outcome.

### Outcome variables

The main outcomes of interest were the MCS at baseline, 1 year after THA, and 1-year change. The MCS ranges from 0 to 100, with higher scores indicating better outcomes. We calculated the change as the absolute difference in MCS scores between baseline and 1 year after THA. The mean ± SD population value in this geographic study area [31] was 46.3 ± 10.1.

### Predictor variables and covariates

The main predictors of interest included the WOMAC pain and function scores at baseline, 1 year after THA, and the 1-year change. We calculated differences in WOMAC pain and function scores as the absolute difference between baseline and 1 year post-op pain and function scores, respectively. We took into consideration participants’ age, sex, body mass index (BMI: < 25, 25–29.9, 30–34.9, 35.0+), education level (< 9, 9–12, > 12 years of education), insurance status (private or public), smoking status (ever or never), medical co-morbidities such as diabetes (yes or no), the American Society of Anesthesiologists (ASA) score (1 = normal healthy patient, 2 = patient with mild systemic disease, 3 = patient with severe systemic disease, or 4 = patient with severe systemic disease that is a constant threat to life) [32], medications used including antidepressants or opioids, Charnley disability grade (A = involving one hip, B = involving both hips, or C = multiple joints or other disabilities leading to difficulties in ambulation) [33], and reason for THA (primary vs. secondary OA, the latter including dysplasia, inflammatory arthritis, aseptic necrosis or post-traumatic origin).

### Data collection

Preoperatively, the questionnaire was sent out to all patients undergoing elective THA approximately 10 to 14 days prior to surgery. The follow-up questionnaire was sent out 1 year after surgery to all patients still alive. For patients who did not return their 1-year questionnaires, another follow-up questionnaire was sent about 3 months after the first mailing. Information on the baseline characteristics including age, sex, and insurance status was recorded at the time of admission. Reason for OA and Charnley disability grades were recorded on a pre-specified form by the operating surgeon. Medical co-morbidities, medication use at the time of admission, BMI, ASA score and smoking status were obtained from the anaesthesia report and discharge summary. Information on education level was obtained from the patient via the preoperative questionnaire.

### Statistical analysis

Regarding the first aim (to examine the change in mental health from before to 1 year after surgery and to identify variables associated with improvement), we calculated means and standard deviations (SD) for baseline, 1 year after surgery, and 1-year change in MCS scores overall, and by subgroups defined by age, sex, BMI, education, insurance, smoking status, ASA scores, diabetes, antidepressant or opioid use, Charnley scores, and OA status. To show the magnitude of the overall 1-year difference, we estimated Cohen’s effect size where 0.2, 0.5, and 0.8 were considered respectively as small, medium, and large differences between baseline and 1 year after THA [34]. We also plotted the distributions of the baseline and 1 year after THA MCS scores, using kernel density plots.

Regarding the second aim (to examine the association between change in mental health and change in pain and function over time), we calculated means and standard deviation (SD) for baseline, 1-year, and 1-year change in WOMAC pain and function scores. We then examined 1-year change in MCS by quartiles of 1-year changes in pain and function. We also used 2 separate linear regression models to predict 1-year change in MCS as the main outcome of interest, one model with 1-year change in pain and the other with 1-year change in function as the main predictor of interest. We performed both unadjusted linear regression, and adjusted for education, age, BMI, sex, smoking status (ever vs. never), insurance status (private vs. public), diabetes, ASA score (1 vs. 2+), and Charnley score (C vs. A and B), OA status (primary vs. secondary). By visual inspection, the distributions of regression residuals of these models were reasonably bell-shaped. Finally, as education level was an important covariate and approximately one fourth of our participants were missing education data, we further performed sensitivity analyses using a simultaneous multiple imputation for the education level. In brief, we entered education, age, and BMI as continuous variables in addition to sex, insurance, tobacco, ASA, diabetes, hypertension, and Charnley score into the model with 1-year change in pain or function predicting 1-year change in MCS score to perform multiple imputation of missing data for multivariable adjusted linear regression. We used IBM Windows SPSS V.22 (IBM Corp., Armonk, NY, USA) for all statistical analyses.

## Results

We included 610 participants in the study. Of those, slightly over half of them were women, 1/3 of participants were younger than 65 years, 23% were overweight or obese, 26% had less than 9 years of education, 85% had public insurance, and 2/3 never smoked. Moreover, approximately 10% of participants had diabetes, used antidepressants, used opioids at baseline, or had THA for reasons other than primary OA.

**Fig. 1:**
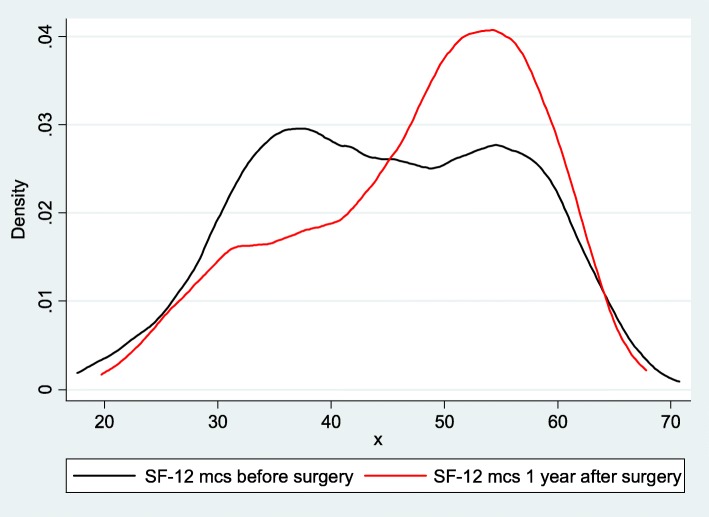
Kernel density plot of SF-12 mental component scores (MCS) before and 1 year after primary total hip arthroplasty

Regarding the first aim, MCS scores prior to THA had an almost uniform distribution between values of 30 and 60 (Fig. 1). One year after THA, MCS scores were unimodal with highest density at 55. The overall mean (SD) MCS was 44.7 (11.2) at baseline and increased to 47.5 (10.5) 1-year post-THA (Table 1). The average 1-year change was 2.8 (95% CI: 1.9, 3.6), for an effect size of 0.26 and is significantly different from 0. Subgroups with 1-year change in MCS scores of 2.8 (equivalent to the mean overall 1-year change) or higher included those: younger than 55 or 75 years and older; women; BMI < 25, or 30 and greater; having a high school education or less; having public insurance; being a smoker; having ASA scores 2 or higher; having diabetes; having used opioids; having a Charnley A or C classification; and having secondary OA as diagnosis. However, group differences were statistically significant for education and ASA scores and borderline statistically significant for insurance type. Moreover, there was no statistically significant difference in 1-year change in MCS by baseline antidepressant use or opioid use subgroups.

**Table 1:** Mean and Standard Deviation (SD) of Mental Component Scores at Baseline, 1 year after Total Hip Arthroplasty, and 1-Year Change Overall and By Baseline Characteristics

Baseline characteristics	N (%)	Baseline	1 year after THA	1-Year Change	*P*-Value for Differences in 1-Year Change by Groups
Overall	610	44.7 (11.2)	47.5 (10.5)	2.8 (10.8)	
Age: <55	81 (13%)	45.6 (10.8)	49.0 (9.6)	3.4 (10.6)	0.940
55–64	123 (20%)	43.9 (11.9)	46.6 (11.1)	2.7 (10.7)	
65–74	191 (31%)	46.2 (11.6)	48.7 (10.7)	2.5 (10.9)	
≥75	215 (35%)	43.6 (10.5)	46.4 (10.2)	2.8 (10.9)	
Sex: Women	323 (53%)	43.1 (11.2)	46.2 (10.9)	3.1 (11.0)	0.425
Men	287 (47%)	46.6 (11.0)	48.9 (9.9)	2.4 (10.6)	
BMI: <25	224 (37%)	46.4 (11.0)	49.2 (9.9)	2.8 (10.7)	0.288
25–29.9	248 (41%)	44.5 (11.5)	46.5 (10.4)	2.0 (10.8)	
30–34.9	104 (17%)	42.6 (10.4)	46.4 (10.9)	3.8 (11.1)	
≥35	34 (6%)	42 (11.6)	47.1 (12.9)	5.1 (10.9)	
Education: Missing	154 (25%)	44.3 (11.6)	46.1 (11.3)	1.8 (11.0)	0.045 [Tukey’s pairwise test: < 9 vs. ≥13 Yrs = 0.078]
<9 Yrs	160 (26%)	41.3 (11.3)	45.5 (10.7)	4.2 (11.5)	
9–12 Yrs	140 (23%)	44.4 (10.8)	48.1 (10.2)	3.7 (10.6)	
≥13 Yrs	156 (26%)	49 (9.8)	50.3 (9.2)	1.3 (9.7)	
Insurance: Private	94 (15%)	49.7 (9.8)	50.5 (9.5)	0.8 (10.7)	0.058
Public	516 (85%)	43.8 (11.2)	46.9 (10.6)	3.1 (10.8)	
Smoking: Never	383 (64%)	45.4 (11.1)	47.5 (10.5)	2.1 (10.9)	0.080
Ever	219 (36%)	43.6 (11.4)	47.2 (10.5)	3.7 (10.5)	
ASA score: 1	74 (12%)	49.8 (10.1)	49 (9.7)	−0.9 (9.6)	0.005 [Tukey’s pairwise test: 1 vs. 2 = 0.008 1 vs. 3–4 = 0.006 2 vs. 3–4 = 0.603]
2	448 (73%)	44.3 (11.3)	47.4 (10.6)	3.1 (10.8)	
3–4	88 (14%)	42.6 (10.4)	46.8 (10.8)	4.3 (11.1)	
Diabetes: Yes	58 (10%)	42.7 (11.8)	46.8 (9.7)	4.1 (10.2)	0.312
No	552 (90%)	44.9 (11.1)	47.6 (10.6)	2.6 (10.8)	
Antidepressant: Yes	60 (10%)	39 (9.4)	41.8 (11.0)	2.8 (12.4)	1.0
No	550 (90%)	45.4 (11.2)	48.1 (10.3)	2.8 (10.6)	
Opioid: Yes	82 (13%)	41.4 (11.6)	44.7 (11.1)	3.3 (10.6)	0.639
No	528 (87%)	45.3 (11.1)	47.9 (10.3)	2.7 (10.8)	
Charnley: A	257 (42%)	45.8 (11.2)	48.7 (9.4)	2.9 (11.1)	0.138
B	150 (25%)	45.6 (11.5)	47 (10.9)	1.4 (10.4)	
C	203 (33%)	42.7 (10.9)	46.4 (11.3)	3.7 (10.6)	
Primary OA: Yes	543 (89%)	44.7 (11.3)	47.4 (10.5)	2.7 (10.8)	0.721
No	67 (11%)	45.4 (10.5)	48.6 (10.5)	3.2 (11)	

Regarding the second aim, the following are descriptive statistics for WOMAC pain and function, and 1-year change in MCS scores by quartiles of 1-year changes in pain and function. For WOMAC pain, mean (SD) at the baseline was 39.6 (18.3), and at 1-year post-THA it was 83.8 (20.4). The change in pain from baseline to 1-year post-THA was 44.2 (95% CI: 42.4, 46.0). For WOMAC function, mean (SD) at baseline was 40.2 (18.8), and at 1-year post-THA it was 78.3 (22.1). The change in function from baseline to 1-year post-THA was 38.1 (95% CI: 36.2, 40.0). Results for the change in MCS scores by quartiles of WOMAC pain and function improvement indicated that mental health improvement was greatest in the patients with the most improvement in pain or function 1-year post-THA. In fact, the increasing trends in MCS change with a decrease in pain or increase in function were both statistically significant (Table 2**)**.

**Table 2:** SF-12 Mental Component Scores at Baseline, 1 year after Total Hip Arthroplasty, and 1-Year Change by quartiles of WOMAC pain and function improvement

	N	SF-12 MCS Prior to THA Mean (SD)	SF-12 MCS 1 year after THA Mean (SD)	SF-12 MCS 1-Year Change Mean (SD)
Improvement WOMAC pain in quartiles				
<30	182	44.2 (11.1)	45.1 (10.8)	0.8 (10.2)
30–44.9	128	46.8 (10.7)	49.1 (9.9)	2.2 (10.5)
45–59.9	163	46.4 (10.3)	49.4 (9.6)	3.0 (10.7)
≥60	128	41.4 (12.5)	46.8 (11.2)	5.5 (11.5)
Total	601	44.7 (11.3)	47.5 (10.5)	2.8 (10.8)
p-value for linearity (ANOVA)				<0.001
Improvement WOMAC function in quartiles				
<21	128	42.3 (10.7)	42.8 (11.0)	0.5 (10.4)
21–38.9	155	46.2 (11.5)	47.6 (10.4)	1.4 (9.3)
39–53.9	157	46.4 (10.8)	49.4 (9.1)	3.0 (11.4)
≥54	145	43.5 (11.4)	49.1 (10.5)	5.7 (11.0)
Total	585	44.7 (11.2)	47.4 (10.5)	2.7 (10.7)
*p*-value for linearity (ANOVA)				<0.001

Results from multivariable analysis showed that improvements in pain and function were strongly associated with improvements in mental health (Table 3). On average, a 10-point difference in the 1-year change in pain score was associated with a 0.78 (95% CI: 0.40, 1.16) point difference in the 1-year change in MCS after controlling for covariates. The corresponding change in function was associated with a 0.94 (95% CI: 0.56, 1.32) point increase in 1-year change in MCS after controlling for covariates. Results from multiple imputations were very similar for WOMAC pain and function and between values for the crude and adjusted estimates.

**Table 3:** Predicting 1-Year Change in Mental Component Scores Results from Multiple Linear Regression

Per 10 Unit Change	Unadjusted Beta (95%CI)	Adjusted^a^ Beta (95%CI)	Adjusted Multiple Imputation^b^ Beta (95%CI)
WOMAC Pain	0.83 (0.45–1.21)	0.78 (0.40–1.16)	0.80 (0.42–1.18)
WOMAC Function	0.95 (0.58–1.32)	0.94 (0.56–1.32)	0.96 (0.59–1.33)

^a^Adjusted for BMI, Age, ASA (C vs. Oth), Insurance (private vs. public), Primary OA, smoking, Charnley (C vs. oth)

^b^Adjusted using simultaneous multiple imputation (included variables: Education, BMI, Age, ASA (C vs. oth)

## Discussion

Mental health improved from baseline to 1 year after THA. The observed change in mental health was similar in patients with and without antidepressant use. The magnitude of the change in mental health was strongly associated with the degree in improvement of pain and function. Moreover, alleviating pain and improving function was associated with improved mental health after taking into account differences in baseline characteristics.

We are not aware of any previous publication evaluating the associations between pain and function improvement and mental health gain after THA. Previous studies have assessed quality of life before and after THA and they have included MCS scores among other instruments [14–19], [20–22] The results from these studies concur that MCS improved after THA. The degree of improvement ranged from small to moderate effect sizes, which is in accordance with our findings.

Regarding the link between pain and depression, a previous study of Canadian patients suggested that pain may have an indirect effect on depression via its effect on disability [8]. In another Canadian study, the effect of physical health on self-rated health was mediated by mental health [9]. While we cannot make conclusive causal inference on whether pain alleviation improves mental health status by improving function as a result of THA as suggested by Hawker et al. [8], our study implies that both pain alleviation and improvement in function is associated with improved mental health, controlling for potential confounding by baseline characteristics.

The strengths of our research study included the representativeness of our study population with that of the surrounding Swiss population [35], especially with regard to MCS [31]. Moreover, the instruments we used to collect information on patient-reported outcomes have been validated and widely used [29]. Regarding possible limitations of this study, level of education was missing in about 25% of our study population. While we cannot rule out that education may not be missing completely at random, the reason for missing education data was that we did not begin collecting education information until 2012. This may impact power more than validity of our estimates or the inference of our study results, as confirmed by results from the sensitivity analysis of imputed data. Another type of missing data that we cannot account for is loss of follow-up due to deaths. However, loss to follow due to mortality remains low given the short duration of follow up after surgery.

## Conclusions

In conclusion, mental health improved from baseline to 1-year post-THA. The degree of improvement in mental health was strongly associated with the degree of improvement in pain and function, taking into account potential confounders. Thus, improvement in mental health can be an important benefit of surgical intervention. As a consequence, depression or mental health distress frequently seen in patients with osteoarthritis may be successfully altered by hip replacement surgery.

## Data Availability

The Geneva Arthroplasty Registry obtains patient consent for data collection and protects access to the data. We have established data use procedures through our Publication and Ancillary Studies Committee. Investigators can formally request analytic access to data through these mechanisms.

## Abbreviations

THA: total hip arthroplasty
TKA: total knee arthroplasty
mTJA: total joint arthroplasty
OA: osteoarthritis
MCS: mental component score
WOMAC: Western Ontario McMaster University Osteoarthritis Index
SD: standard deviation
BMI: body mass index
SF-12: 12 Item Short Form Survey
ASA: American Society of Anesthesiologists
